# Supplementary material for: Molecular Cloning, Characterization, and Expression Regulation of Acyl-CoA Synthetase 6 Gene and Promoter in Common Carp Cyprinus carpio
Source: Int J Mol Sci. 2020 Jul 3;21(13):4736. doi: 10.3390/ijms21134736 (PMC7370118; doi:10.3390/ijms21134736)
Supplement: Supplementary file 1 [file ijms-21-04736-s001.pdf]

	CTATATATACAGAATATTGGTGAACAACAAAGCAGGCTTTTAGTTTGGGCTGCTTTTGGA
	TCTAAATATGTATTTTGCTGCTGTGGGTGCGTAACGGCTCTCATCCAGTAGCCCTTGTCC
	TTTTTCATATAATGTGGAGAGATGTGGGAGACTGTGAAGTGTTGGACTCTTATGATGATG
181	CTGATGGCAGTGAATCTGTGTGTCTCTCACAGCTATGCAGTTTCAGGATTGGTTACGTTC
1	M Q F Q D W L R S
241	CTTGCGTTTCACTGGAGGAGATGGCAAGTCTGAAATGGACAATCTGAAGACGCTCCTTCC
10	L R F T G G D G K S E M D N L K T L L P
301	GTCTCTGCCCACGTCCTTCTCTCTGTCCTCTCTCTCCCTTTCTCCGTCCTCTATCATAGG
30	S L P T S F S L S S L S L S P S S I I G
361	CTTAGGAGCGCTAGCCTCTCTCACAACCTACTGGCTGGTGACCAGACCTCGACCAACCCA
50	L G A L A S L T T Y W L V T R P R P T Q
421	GCCTCCATGTGACCTACAGGCTCAGTCCATACCTGTGCAGGGAGATCAGAGCTGTAGACG
70	P P C D L Q A Q S I P V Q G D Q S C R R
481	CTCCGCTTTACTGACGGATGACAATCTGCTGGAGTTTTACTATGAGGACACAAAGACAGT
90	S A L L T D D N L L E F Y Y E D T K T V
541	GTACGACATGTTCCAGAGAGGACTGCAGATAGCAGGCAATGGTCCTTGTCTTGGCTTCAG
110	Y D M F Q R G L Q I A G N G P C L G F R
601	GAATCCCGGAGAGCCCTATCAGTGGATCTCTTACACTGAGGTCTCAGAAAGGGCACAGGT
130	N P G E P Y Q W I S Y T E V S E R A Q V
661	GTTGGGATCAGGTTTGCTGGCGAAGGGTTGCAAACCAAACCCACAGCAGTTTGTGGGCAT
150	L G S G L L A K G C K P N P Q Q F V G I
721	CTTTGCTCAAAACAGACCAGAGTGGGTCATTGCAGAGCTGGCCTGTTACACCTTCTCTAT
170	F A Q N R P E W V I A E L A C Y T F S M
781	GGCTCTGGTGCCTCTGTACGATACTCTTGGATTAGAGGCTATGGTTCACATCCTTAACCT
190	A L V P L Y D T L G L E A M V H I L N L
841	GGCGGAGATCTCTATGGTGATCTGTGATAAGGAGGATAAGGCAGAATCTCTGTTGAAGCA
210	A E I S M V I C D K E D K A E S L L K H
901	TAAAGAGAAGGGAGTGACTCCCGCTCTCTCCTGTCTGGTGCTCTTTAACCCCTTCAGCGC
230	K E K G V T P A L S C L V L F N P F S A
961	TGCTCTGCTGGAGAGAGGAAGGAAGTGCGGTGTGGAGATACTACAGCTGTCACAGATTAT
250	A L L E R G R K C G V E I L Q L S Q I M
1021	GGATCTAGGAAGAGAAAATTTAAAGCCACCTGTGCCTCCTAAACCTCAGGACCTGGCTAT
270	D L G R E N L K P P V P P K P Q D L A I
1081	GGTGTGTTTCACCAGTGGGACCACAGGAAAGCCTAAAGGAGCCATGATCACTCACGGCAA
290	V C F T S G T T G K P K G A M I T H G N
1141	CATCGCCTCCAACACCTCCTCTGTCATCAAGATCCTTGAGGGCTACTTTGTGATTCGTCA
310	I A S N T S S V I K I L E G Y F V I R Q
1201	GGAGGATGTGTCCATCTCCTATCTGCCACTCGCTCACATGTTTGAGCGTATGATCCAGGT
330	E D V S I S Y L P L A H M F E R M I Q V
1261	GTCGATGTTTTGTCATGGAGCAAGGGTTGGTTTTTACCAGGGTGACATCTCTCTGCTGAT
350	S M F C H G A R V G F Y Q G D I S L L M
1321	GGATGACATAAAGACCCTGAAGCCCACCTTCTTCCCCGTGGTTCCTCGATTACTCAACCG
370	D D I K T L K P T F F P V V P R L L N R
1381	CATCTATGATAAAATTGTGGGCTCGGTTACGTCTCCGCTGAGGAGAGCCCTTCTTCATTA
390	I Y D K I V G S V T S P L R R A L L H Y
1441	CGCCGTGAGGAGGAAACAGGCCGAGCTGAGCAGTGGAGTCGTGAGGAACAACAGCGTGTG
410	A V R R K Q A E L S S G V V R N N S V W
1501	GGACCGACTGATCTTCAATAAGATACAGGCCAGTCTGGGTGGAAACCTGCGATTCATCCT
430	D R L I F N K I Q A S L G G N L R F I L
1561	CACTGCATCCGCTCCGATCTCTCCGACTGTCCTGTCCTTCCTCAGAGCCACCCTCGGCTG
450	T A S A P I S P T V L S F L R A T L G C
1621	TCTGATTTTCGAGGGTTATGGACAGACGGAGTGCACTGCAGGATGTACCTTTTCTATGCC
470	L I F E G Y G Q T E C T A G C T F S M P
1681	TGGTGACTGGAGTGCAGGTCATGTCGGCGCTCCTTTACCCTGTGCTATGGTGAAGTTAAC
490	G D W S A G H V G A P L P C A M V K L T
1741	CGACATCCCTGACATGAACTACTACGCCAAGAACGGAGAAGGAGAGGTCAGTATCCGGGG
510	D I P D M N Y Y A K N G E G E V S I R G
1801	TCACAGCGTCTTCCGAGGGTATCTGAAGGATGAGGAGAGGACGGGAGAGACGCTGGACGC

```
530     H  S  V  F  R  G  Y  L  K  D  E  E  R  T  G  E  T  L  D  A
1861   TGATGGCTGGCTTCACACTGGAGATGTGGGACAGTGGCTGCCGAACGGGACTTTGAACAT
550     D  G  W  L  H  T  G  D  V  G  Q  W  L  P  N  G  T  L  K  I
1921   TATGGACAGGAAGAAGCACATCTTTAAGCTGTCCCAGGGCGAGTACATTGCTCCTGAGAA
570     I  D  R  K  K  H  I  F  K  L  S  Q  G  E  Y  I  A  P  E  K
1981   GATCGAGAACGTCTACACTCGCTGTGTTCCTGTTCTCCAGGTCTTTGTGCACGGAGATAG
590     I  E  N  V  Y  T  R  C  V  P  V  L  Q  V  F  V  H  G  D  S
2041   TCTACAATCTTATCTGATAGGAGTTGTAGTTCCAGACCCGGAGGTGTTTGTCGACTGGGC
610     L  Q  S  Y  L  I  G  V  V  V  P  D  P  E  V  F  V  D  W  A
2101   CAAAGAAAGAGGGATCGTTGGGTCATATGAAGAGCTTTGTCAAAATCCTGATGTGAAGAA
630     K  E  R  G  I  V  G  S  Y  E  E  L  C  Q  N  P  D  V  K  K
2161   AGCCGTGCTGGAGGACATGACGGCAGTGGGGAAGGAGGCAGGACTGAAGTCATTTGAGCA
650     A  V  L  E  D  M  T  A  V  G  K  E  A  G  L  K  S  F  E  Q
2221   GGTGAAGGACTTATATTTGCACCCTGACATGTTTAGCGTGTCAAACGGCCTCCTCACGCC
670     V  K  D  L  Y  L  H  P  D  M  F  S  V  S  N  G  L  L  T  P
2281   GACCCTGAAGAGCAGGCGTGTGGATCTCAGGCGAGTCTTCAGCGAGCAGATCGCACGGAT
690     T  L  K  S  R  R  V  D  L  R  R  V  F  S  E  Q  I  A  R  M
2341   GTACAGGAAATCATCCGTATAGACCACATCACATTTACAAAGCCATTTTCTCTAAAACAG
710     Y  R  K  S  S  V
       TAGAACTAACTTAGCCAGATGCTACTGACAGGAAACTGTTCTCCATAATTTATGATAAAT
       CATCTCGAAGACTCAAGTCATGTAGGGATCTAGATTGTGACAGACACTCCATCCTAATAC
       ATAGTTCAGGAATATTTAGTTGCATCTTCCAAGATTTGGGAACTGAGGAACTTATTTTAA
       TGAATAGTCTTACGATCAGCGTCTTAGATATTTTACAGTTTTGTAAAGAACAATCTGTAT
       GAATTGTAATGCTTTGTTTCATTTGTTGTGTCGACAGATGAAATGACTGTTGGACAGTCT
       GTAAATGAGTGTTTTCAAATGTAAATGTTTGACTAATTGCAGCTGATAGAGATTTTACTG
       TATTTTACTGACTGATGCCTTATTACAGTTTTTCTTTTTCATACCAATCACAGCTCTAAC
       TGTTGTTGAAGCACAGATCTGATTGGTTCATGTGGATGTCACTCAGGAAACTTTGTACTA
       TGGTGAAAGGCATTAGGATGTTGATCTGTTGCTTACAAGAAAACCTTACTGGTTCATTCC
       AGAGTAAATCACAATCCTGCCCAGACTACAATACGTGCACTAGACGTCATTTTGGATGAA
       AAAGGCGAGATAAATGAGTTGATTTCAATTAAAATCAAGAATTTTCAGACACTGAATATC
       CACAACCAATTTACTGTAAGAATGTTTGACAGGTTAACACAACAGCAAACAATTTGTTTA
       AAGGAAAACAATTTTACAGACGAAAAATGCACACATTTTTGGGAATCGTAAGGCTGTGGT
       CCAGAAAATTCTGTGGTTTTCGATTATTTCAGAAGTCAAACATGCATTTATTTTAAAAGC
       AAAGCAGTAAAAACGTTGTATAATGCTTACATTAAGTTGCTTGGTAGAGAAAGGCACTGA
       ATTTGCTTACACTGGACTATAGTGAACAACATTTTAAATAATGTCAAACCGAACCAATAA
       TCTTTTGTTTATACAATTAAGGCTTTTCCAGTTTAAAAACTTCACTACATCGTGATTGTC
       AGTAACTGATGCTATTCACATCAACGAGATGAAAATGTGTGGCTTAAATAATTGGCCCTT
       GTGTATTATGGCTGTAAGACTACTGGTTAATGTATATAAACCGGTCTTTCCTTCATTACA
       AACACAATGAGCACAAACACAGGTCAGACACAAAAACTGTAAAATGTTTCACGTGTGTCA
       GTAGAGGGAGGCAGAGTATCATTTAGATGCATGTATTTCATCACAGAATCCACACCAGAC
       TAACATGATTTCTGTTACCTGACGATCGCACTGTAACATTTTTCCAACAATTCTCTGCAC
       TATTCCAGTGTTACACTGTTTTCTGAAGATACTGTAAAAGCATAAAAAAAAAAAAAAAA
```

Fig. S1. The complete cDNA sequence and predicted amino acid sequence of common carp *acsl*6

TGCCCAGAATTTGCACAGATTACGTGTTGTTATTACTATTATTTAAAAATAAATACTTCT	−1730
TGTAATTGATGGGCTTGTTGTTCAGTATTTCAACAAATTAGATGTACATTTGAGATATTT	−1670
TTTTGCTTTTCATTGCAAACTATAGACTAACTGAGTTTAATTATTTCATTTGCATTTATA	−1610
ATGTTGCTGTTATGACATATCTACAATTAAAATATGGGCAATTAAATATGTAAATATTCA	−1550
AATGCCTGTACATGAATAATAGAAAACAAATTTCCTTATGATGAATGTCTTTTTACTGTC	−1490
TGTCATTGTTATTCAACTAAATGTATAAAACAAATAAAATGTATAATATTAATAAGTAGT	−1430
TTATCAAAAATCATTTAGACAGTCTACCGGAACCACATCAAGTGACAGACCTGGCTGGGA	−1370
TATGCTTATCAGTGTCTTCTCTGAAAGTTATTTTAACATTCTGATCATATGATTCCCTAG	−1310
CTTTTTTTTTTTTATTAGCCTAAATAAGCTGATGAAATACCACACAATGTGAAACATTAT	−1250
GTAAGTTGAATACTTTTACTTAACATAAAAAAATCAACCATGGTTTTACTATTAAAATAA	−1190
ATAAATAAATAGCCTCTCTCTCTCTCTCTCTCTCTCATCTCTCTCTGATATATATAATAT	−1130
ATATCATGTGTGTGTGTGTGTGTGTGTGTGTGTATTATTATTATCATTATTATATAAAAATA	−1070
AATACAAAAAATGTTTACTATGGTTAAACCATGGTAAGTAAACCCTGCTAACATAGTTTA	−1010
GCCATGGTATTTGTACAACTGTGGTTATACGATTGGTAATCAAAACATGTTTACTACATT	−950
TACTATAATAAAATCATGGTTAATTTTCGTAAACATCGTTGAGGTCCCCCTCTGTGGGCT	−890
GTGCGTTTCTATTTTTACAGCACGTTTCTGGTCGTGTTGTGAGTATTTGCAGCGTGTTGC	−830
TATATTTGGTTGTTCTGCGAGTATTTGCAACACGTGCTATCAAACTGATGAAGACGTATT	−770
CTTAATTTGCTGGTGTTTTTCTATTTGCATGTGTTTTAGGTTGCAGCTCTCTCAGCTACC	−710
AGCTAAAAACACATCATCATCATCCTGCATATGCAAAACTTATATCTAAATATGTTACAA	−650
ATTGAAAGTAAATGCACATTATTCTTAAAGTAAGTGGGGCATCTTCCCTCATCTTACCGT	−590
TAAAAATAAATAAATAAATAAATAAATAAATAAATAAATAAATATAAATTGTAGAGTGTG	−530
TCAGGGCTGGTGTTTGACTGCTCTGGTTAACAGCCATTTATTATCTTTGCAATTATATAG	−470
TGGAAAAAAATGGCACATTTCTAACAGCTTTAATAAGGGTTGGTGAGCTGCTATTATTTGG	−410
TGAGCTGCTATTAAAACATATTTGACATTAATAAATAGAATGACAGTGTTGTGATGTTCT	−350
TTGTCTGTGGTTAAAGGTGAAATGTGTCATTTCTATGCCACTATAGTAGCATACAGAATA	−290
GTCATCCTTTTTTTTTTTTTTTTGGCTGCTTGGTATCAGGTTTGCAGATCAGGATAGAAA	−230
TACTTATAGTTTGAATCAGATTTTATTTTCCATTTCATTTTCATTTTAGTTCAAGTTTTA	−170
GTAGTCTAACTAATATCAACAAATGGATCCTTATTGTAAAGTGTTGCATATTCGACTTTT	−110
CTTTTGTGGAATATTAAATAAGATATTTCGAAAGATGCCTTTTTTAATGAATGTCAGTAT	−50
TCATAAAGCCGTTTGGTTACCAACAATTCTAAATGTCTTATTTTGTGCTCTATATATACA	+11
GAATATTGGTGAACAACAAAGCAGGCTTTTAGTTTGGGCTGCTTTTGGATCTAAATATGT	+71
ATTTTGCTGCTGTGGGTGCGTAACGGCTCTCATCCAGTAGCCCTTGTCCTTTTTCATATA	+131
ATGTGGAGAGATGTGGGAGACTGTGAAGTGTTGGACTCTTATGATGATGCTGATGGCAGT	+191
GAATCTGTGTGTCTCTCACAGCT**ATG**CAGTTTCAGGATTGGTTACGTTCCTTGCGTTTCA	+251
CTGGAGGAGATGGCAAG	+268

Fig. S2. Sequence of candidate promoter of *acsl*6 in common carp. Gene sequence is numbered relative to the first base of the transcription start site (TSS), assumed to be the first base of the 5′ non-coding exon. ATG start codon was shown in bold.

Table S1. Ingredients and composition of experimental diets (dry matter basis)

Ingredient	Dietary treatment
Soybean meal	25
Rapeseed meal	25
Cottonseed meal	17
Wheat middling	10
Rice bran	13.2
Vitamin mixture [1]	0.5
Mineral mixture[2]	0.5
Choline chiorlde	0.1
Dicalcium phosphate	0.5
L-Lysine	0.1
DL-Methionine	0.1
Peanut oil	3.67
Perilla oil	4.33
Proximate composition	
Dry matter	86.55
Crude protein	32.10
Crude lipids	8.13
Ash content	6.61

Note: 1. One kilogram of mineral mix containing the following: K 100 g, Mg 30 g, Fe 8 g, Mo 1 g, Zn 30 g, Mn 2 g, Co 1 g, I 500 mg, Se 40 mg. 2. One kilogram of vitamin mix containing the following: A 3×10^4 IU, D3 3×10^4 IU, E 3×10^3 IU, K3 20 mg, B1 20 mg, B2 480 mg, B6 360 mg, B12 100 mg, nicotinic acid 170 mg, calcium pantothenate 80 mg, folic acid 170 mg, biotin 10 mg, inositol 8 g, C-monophopholipid 10 g.

Table S2 Fatty acid composition of experimental diets (% area)

Main fatty acids	Dietary treatment
14:0	1.44
16:0	16.84
16:1	1.09
18:0	3.61
18:1	18.14
18:2*n*-6	23.34
18:3*n*-6	/
20:3*n*-6	/
20:4*n*-6 (ARA)	/
18:3*n*-3	25.44
C18:4n-3	/
C20:4*n*-3	/
20:5*n*-3 (EPA)	/
C22:5*n*-3	/
C22:6*n*3 (DHA)	/

Note: ARA, Arachidonic acid; EPA, Eicosapentaenoic acid; DHA, Docosahexaenoic acid
